# Nature-Inspired Drugs: Expanding Horizons of Contemporary Therapeutics

**DOI:** 10.1155/2019/6218183

**Published:** 2019-05-02

**Authors:** Azhar Rasul, Ghulam Hussain, Zeliha Selamoglu, Maria P. López-Alberca

**Affiliations:** ^1^Department of Zoology, Government College University, Faisalabad, Pakistan; ^2^Department of Physiology, Government College University, Faisalabad, Pakistan; ^3^Faculty of Medicine, Department of Medical Biology, Nigde Omer Halisdemir University, Nigde, Turkey; ^4^Centre for Drug Discovery, Northeastern University, 360 Huntington Avenue, Boston, MA, USA

In this special issue, a total of 61 articles were received and 20 of them were approved for publication. This special issue demonstrated the ever-growing role of natural products as lead structures for the treatment of cancer, microbial infections, oxidative stress-associated health ailments, neurodevelopmental disorders, and polycystic ovary syndromes. Phytochemical investigations of natural products for the exploration of bioactive entities, eco-friendly, rapid, and cost-effective synthesis of drugs from natural products, utilization of green chemistry approaches, and optimization of nature-derived compounds for the synthesis of potent derivatives can build up stronger foundation of nature-inspired drug discovery.

This special issue has successfully attracted various interesting research articles and reviews addressing several aspects of natural product-based drug discovery, nature-inspired synthesis of nanoparticles, synthetic analogues of natural products as novel anticancer agents, cost-effective green chemistry approaches, and nanocarriers for enhancing the natural drug delivery system. For example, M. Azeemuddin et al. have attempted to explore the role of a polyherbal formulation, DXB-2030, to reverse the TP-induced polycystic ovary syndrome in rat models and demonstrated that DXB-2030 has a potential ability to enhance GLUT4 expression, to downregulate testosterone and cystic follicles, thus, recommending its usage for the treatment of polycystic ovary syndromes and inviting other researchers to explore more about DXB-2030's mechanism of action. Cost of the drug should be a key consideration while working on drug discovery. D. A. Jamdade et al. have reported an eco-friendly and low-cost synthesis method of copper nanoparticles from medicinal plants which have capability to inhibit porcine pancreatic *α*-amylase activity and *α*-glucosidase activity, thus, opening up avenues for development of antidiabetic nanomedicine. S. Biswas et al. found 3-hydroxyflavone analogue as a novel inhibitor of epigenetic enzyme, histone deacetylase 8 (HDAC8), which is a therapeutic drug target for cancer. This study also paves a way for further investigations on 3-hydroxyflavone analogue in *in vivo* studies.

This issue also gathered several studies that have explored the capability of various plants for their antimicrobial, antidiabetic, and antimutagenic potential. This screening has identified various novel plants and described their mechanism of action.

Furthermore, this special issue has also published few interesting review articles addressing various aspects of phytochemicals such as pharmacological profile, therapeutic potential, current status in drug discovery, and efficient drug delivery by plant-based nanocarriers. For example, S. Chanda et al. reviewed various nutraceuticals having therapeutic potential and provided the classification of nutraceuticals based upon their mechanism of action, chemical nature, and food availability. However, M. Gharbavi et al. have discussed diversified structures, synthesis approaches, techniques for characterization, and routes of administration of noisome to overcome the blood-brain barrier for the development of efficient drug delivery systems.

Therefore, this issue will hopefully pave a way for researchers and encourage the scientific research community for further research in this field for the development of safer, selective, and cost-effective drugs from natural products.

